# SPOUSAL SUPPORT AND PERCEIVED RESPONSIVENESS IN THE LINK BETWEEN DAILY RUMINATION AND SLEEP: FINDINGS FROM THE NSDE

**DOI:** 10.1093/geroni/igad104.2198

**Published:** 2023-12-21

**Authors:** Sam Molli, Stephanie Wilson

**Affiliations:** Southern Methodist University, Dallas, Texas, United States; Southern Methodist University, Dallas, Texas, United States

## Abstract

The Cognitive Model of Insomnia suggests that increased cognitive arousal may negatively influence sleep. Prior work has supported this theory, indicating that both negative affect and rumination may be negatively related to sleep. However, recent empirical evidence suggests that social support may buffer this impact. Yet, much of the current literature focuses on rumination as a trait characteristic. We set out to investigate both between and within-person rumination and its relation to sleep and negative affect. We also tested whether support, both perceived and received, may buffer the relation between rumination and outcome variables. The sample consisted of partnered participants (n=612, Mage= 62, SD=9.73) from the National Study of Daily Experiences in the MIDUS project. Participants reported daily levels of sleep, rumination, negative affect, and received support; as well as perceived partner responsiveness (PPR) and depressive symptoms at baseline. In multilevel models that controlled for sex, age, race, education, and depressive symptoms and included random person-level intercepts, a significant interaction arose between daily rumination and PPR on daily negative affect (b = -0.0061, SE = 0.0021, p = .004), such that participants reporting higher PPR reported a weaker association between daily rumination and negative affect. Trait or daily received support was not a significant moderator of any outcome and PPR did not significantly moderate the relation between rumination and sleep. These findings further add to our understanding of the distinctions in type of support from a partner. Moreover, this suggests that knowing support is available may buffer distress from rumination.

